# Nationwide survey of importance-performance analysis on infection control and prevention practices at the tertiary hospitals in South Korea

**DOI:** 10.1017/ash.2025.124

**Published:** 2025-09-03

**Authors:** Yeon Hee Woo, Jae Sim Jeong, Hye Ran Choi, Jae Geum Ryu

**Affiliations:** 1 Korea Disease Control and Prevention Agency; 2 Ulsan University; 3 DongA University

## Abstract

**Objectives:** This study was conducted to define the classification of the infection control and prevention (IPC) practices through task analysis method, and to perform importance-performance analysis (IPA) on IPC tasks, consequently providing operational guidelines for the department of IPC. **Methods:** To define the tasks of the IPC practices, the draft was developed through the legal and literature reviews, and the final IPC tasks were confirmed by content validity test. The IPC tasks were assessed by IPA method. The national surveys were conducted by the institutional and individual level from October to November, 2023. The institutional questionnaire was distributed nationally to the IPC director/manager of the tertiary hospitals, and IPC practitioners assessed the IPA of IPC task. **Results:** Two thirds (30/45) of the IPC director/manager responded the institutional questionnaire. A total of 135 IPC practitioners (32 physicians and 103 nurses) completed the IPA survey. The average beds of hospitals participated in this study were 1,060±436. The ICP staffing met the legal requirements in all hospitals. The IPC tasks were consisted of 11 categories and 38 items including surveillance and ICP planning. According to IPA, at a 1^st^ quadrant (high in frequency and importance) surveillance, infectious patient management, health management and action planning of healthcare associated infection were placed. At a 2^nd^ quadrant (high in importance but low in frequency), annual IPC planning was positioned. At a third quadrant (low in frequency and importance), ICP training, policy-making, general hygiene, and ICP staffing were placed. There was nothing at a 4^th^ quadrant (high in frequency but low in importance). **Conclusion:** Most of IPC tasks are consistent in degree of importance and frequency except IPC planning. Based on IPA, priority-based task distribution should be considered to maximize the work efficacy and effectiveness.

